# Match outcome influences accumulated but not relative external load in rolling small-sided games with loser rotation

**DOI:** 10.1186/s13102-026-01821-7

**Published:** 2026-06-24

**Authors:** Terje Dalen, Martin Stallvik, Pål Lagestad, Simen R. Sandmæl

**Affiliations:** Department of Physical Education and Sport Science, Faculty of Education and Arts, Nord University, Levanger, Norway

**Keywords:** Soccer, GPS tracking, Locomotor demands, High-speed running, Training monitoring

## Abstract

This study investigated whether match outcome influences external training load in a rolling three-team small-sided game (SSG) format in semi-professional male football players. Twenty outfield players contributed valid GPS data across five training sessions consisting of repeated 6v6 rolling SSG bouts, where the winning team remained on the pitch and the losing team rotated out. External load variables, total distance (TD), accelerations (ACC), decelerations (DEC), and high-speed running (HSR) were measured using 10 Hz GPS and analysed using repeated-measures ANOVA with Bonferroni corrections, considering both absolute and time-normalised (per-minute) values. Accumulated total distance (TD; F = 18.04, *p* < .001, η^2^p = .52), accelerations (ACC; F = 6.05, *p* = .006, η^2^p = .27), and decelerations (DEC; F = 8.11, *p* = .001, η^2^p = .34) differed significantly between match outcomes, with values decreasing from win to draw to loss conditions. Accumulated high-speed running (HSR) did not reach statistical significance (F = 3.74, *p* = .055, η^2^p = .19). No significant effects were observed for any time-normalised variable (TD: F = 1.57, *p* = .223, η^2^p = .09; ACC: F = 0.49, *p* = .620, η^2^p = .03; DEC: F = 1.72, *p* = .196, η^2^p = .10; HSR: F = 0.45, *p* = .642, η^2^p = .03). These findings indicate that match outcome was associated with accumulated, but not time-normalised, external load in this rolling SSG format. The winner-stays rule should therefore be considered when interpreting accumulated external load in such training formats.

## Introduction

Football performance depends on the integrated expression of technical, tactical, psychological, physiological and physical capacities [1]. Modern match-play is characterized by repeated high-intensity actions such as accelerations, decelerations, changes of direction and high-speed running, which are decisive for performance outcomes and strongly associated with fatigue and injury risk [1–3]. To manage these increasing physical demands, the systematic monitoring and management of training load has become central to optimizing performance while reducing the risk of maladaptation and injuries.

Training load is commonly categorized into internal and external dimensions [4]. Internal load reflects the psychophysiological response to exercise, typically assessed through heart rate (HR), blood lactate concentration and rating of perceived exertion (RPE) [4]. External load represents the mechanical demands imposed on the athlete and is commonly quantified via total distance (TD), accelerations (ACC), decelerations (DEC) and high-speed running (HSR) [5]. In modern tracking systems, these metrics are derived from high-frequency GPS position data, which has been shown to provide valid measures of both steady-state running and explosive transitions [6, 7]. Advances in microtechnology now allow simultaneous and reliable monitoring of both dimensions, enabling a detailed analysis of the dose–response relationship during football-specific training and competition [6].

Small-sided games (SSGs) are widely implemented in football training due to their ability to simultaneously develop tactical awareness, technical execution and physical conditioning within an ecologically valid context [8]. A comprehensive systematic review by Hill-Haas et al. demonstrated that SSGs can elicit physiological intensities comparable to, and in some formats exceeding, those observed during competitive match-play [3, 8]. Importantly, the authors highlighted that training intensity is highly sensitive to task design variables, including pitch size, player number, rules, work-to-rest ratio and coach encouragement. Among these variables, manipulation of player number and pitch dimensions appears particularly influential.

Studies have shown that reducing player numbers increases individual technical involvement and physiological strain, while larger formats reduce individual load but increases collective tactical complexity [9–11]. Similarly, rule modifications, such as limiting ball contacts, can significantly increase high-intensity running and physiological stress, bringing SSG demands closer to match-play [12]. Variations in relative pitch area have also been shown to directly influence physical and tactical behavior [13]. Increasing relative pitch area per player results in greater high-speed running and altered team tactical organization [13]. Complementing these findings, larger pitch formats (LSG) have been observed to elicit greater external loads than smaller formats, particularly in sprinting and high-intensity actions [14]. Crucially, players with superior sprint capacity tend to express higher external loads during such games, suggesting that individual physical profiles may influence performance outcomes within specific SSG constraints [14].

Collectively, these studies demonstrate that SSG design profoundly influences internal and external load responses. However, most of the research has focused on structural task constraints (e.g., pitch size, player number, rule manipulation) [8–13], while relatively less attention has been given to the dynamic competitive elements embedded within SSG formats [8]. In applied training contexts, three-team rolling formats are frequently employed due to their high ecological validity and competitive nature. In these formats, two teams compete while a third rests; the winning team remains on the pitch, and the losing team rotates out. Such designs may introduce a physiological and temporal asymmetry, whereby successful teams accumulate longer continuous playing exposure with reduced recovery, whereas losing teams experience more frequent recovery periods. From a load-management perspective, this structure may lead to disparities in training stimuli among players within the same session. Despite these considerations, the specific impact of competitive dynamics within rolling SSG formats remains insufficiently explored.

Although competitive context and match outcome have been shown to influence physiological and perceptual responses in elite sport [15, 16], empirical evidence within football-specific SSG contexts remains scarce. While existing research has established that work-to-rest ratios significantly modulate physiological strain [8], and that contextual constraints affect external load patterns [17], the specific impact of competitive outcome within a rolling three-team format remains unexplored. Furthermore, as distinct external load thresholds can differentiate various training tasks [18], it is crucial to understand how the structural logic of a drill, such as winner-stays rotation, shapes the actual load distribution. To our knowledge, no previous study has systematically examined whether match outcomes within a three-team rolling SSG format influence external load variables.

This omission represents an important gap in the literature. While prior studies have demonstrated that structural SSG constraints modulate physical output, it remains unclear whether the competitive mechanism itself, specifically winning, drawing or losing within a rolling format, acts as an independent determinant of load. Given that such formats are widely used in semi-professional and professional settings, failure to account for outcome-related asymmetries may influence the precision of load equalization strategies. Ultimately, such discrepancies could lead to unintended variations in the individual training stimulus, potentially affecting long-term player development and recovery management.

Therefore, the aim of the present study was to investigate whether match outcome (win, draw, or loss) influences external training load during small-sided games implemented in a three-team rolling format where the losing team rotates out. Specifically, we examined differences in total distance covered (TD), number of accelerations (ACC), decelerations (DEC) and high-speed running (HSR) in semi-professional male players. By quantifying the external load implications of competitive success within an ecologically valid training design, this study seeks to extend the current understanding of SSG load modulation beyond structural task constraints to include the impact of competitive dynamics. Based on the structure of the rolling SSG format, we hypothesized that winning teams would accumulate higher external load due to extended playing time, while relative (per-minute) load would not differ between match outcomes.

## Methods

### Participants

Twenty male semi-professional football players from a third-level Norwegian football club participated in this study. The players had a mean ± SD age of 21.5 ± 4.3 years, a height of 179.4 ± 6.2 cm, and a body mass of 74.9 ± 5.0 kg. Goalkeepers were excluded from the analysis due to the position-specific nature of their role and their differing movement patterns. All players were active members of the same club during the season and participated in regular training sessions under the guidance of their team’s coaching staff. Participants received a detailed explanation of the research project, its procedures, and their rights via an informed consent form. Participation was voluntary, and withdrawal was allowed at any time. All data were securely stored with password protection until the project’s completion and then deleted. All procedures adhered to institutional and national ethical standards. The study was reviewed and approved by the Norwegian Agency for Shared Services in Education and Research (Ref. No. 520987) and was conducted in accordance with the Declaration of Helsinki.

### Procedures

**Fig. 1:**
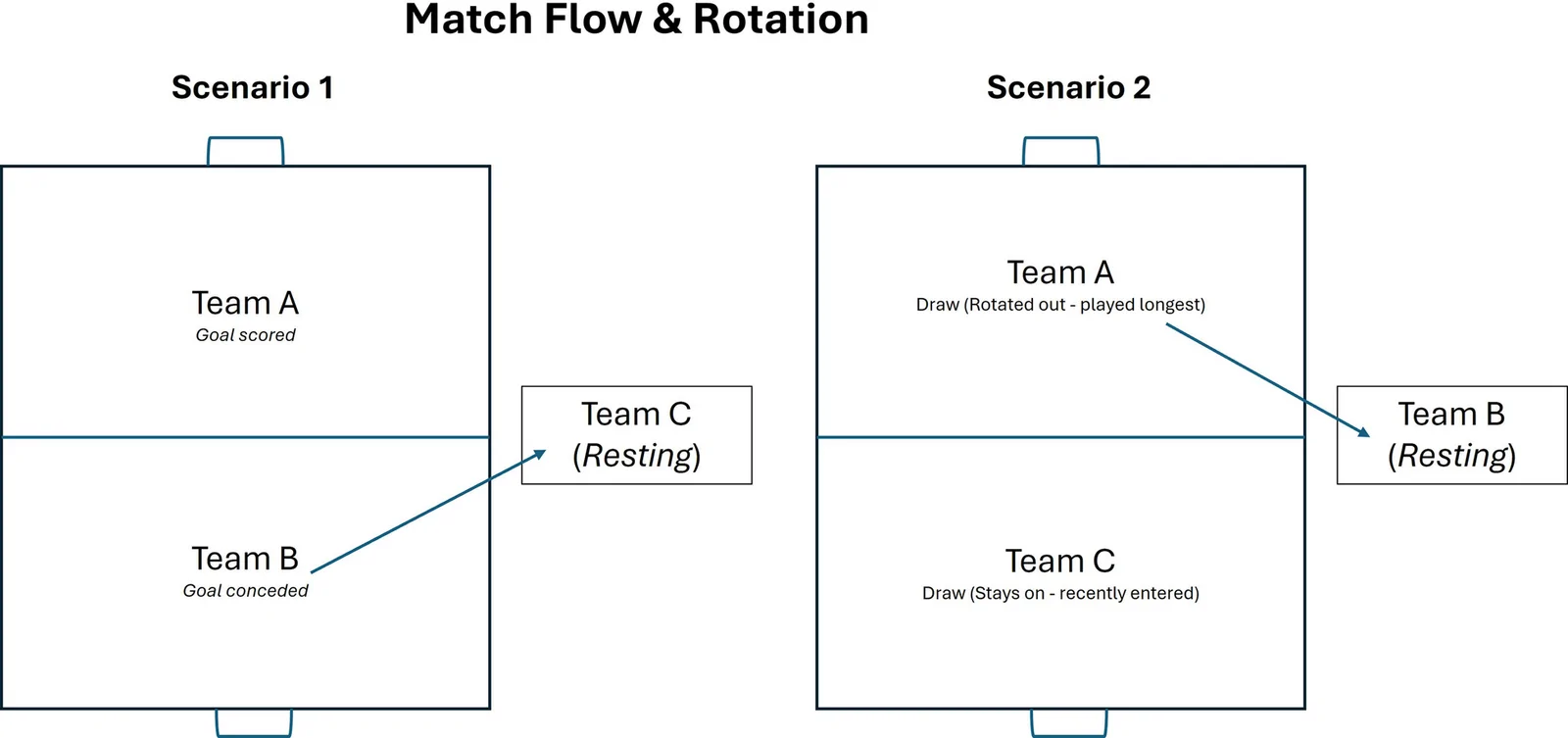
Schematic representation of the rolling three-team small-sided game (SSG) format. Scenario 1: Match flow when a goal is scored, resulting in the losing team rotating out. Scenario 2: Rotation sequence during a draw condition after the 90-s time limit, where the team that has been on the pitch the longest is rotated out while the recently entered team remains

The study employed a repeated-measures design and was conducted across five training sessions during the season. The five sessions were conducted in different weeks but were scheduled at a comparable point within the weekly training microcycle relative to match day. In all five sessions, the rolling SSG protocol was implemented as the final main part of the training session, following the team’s standardised warm-up, a passing drill, and a possession-based exercise. These preceding activities were part of the team’s regular training routine and were led by the coaching staff using the same general structure across sessions. Data were collected under ecologically valid conditions as part of the team’s regular training routines, which were carried out on an artificial turf pitch. Each session included two game-based intervals, each lasting between 12 and 18 min, using a three-team rolling SSG format (6v6). In this setup, three teams of six players, five outfield players and one goalkeeper, competed in 6v6 matches, with a rolling substitution mechanism based on match outcomes. Because of normal training availability, not all 20 outfield players were present at each session. At each individual training session, the SSG format was organised with 15 available outfield players distributed across three teams of five outfield players, each supported by one goalkeeper. Thus, the analytical sample of 20 outfield players reflects the total number of players contributing valid GPS data across the five sessions, rather than the number of outfield players present in each individual session. The teams were formed by the coaching staff for each session to balance playing positions and perceived playing level. The pitch measured 37 × 40 m providing approximately 123 square metres per player. The match ended if a goal was scored, with the scoring team being declared the winner. The winning team remained on the pitch, while the losing team was rotated out. Each match lasted a maximum of 90 s, and if the match ended in a draw, the team that had recently entered continued to play, while the team that had been playing the longest was rotated out (see Fig. 1). On average, nine matches were played during each 12-min period. The typical duration of each match was 67 s, and the average rest period between matches was 20 s. To ensure comparability across sessions, all load-related data were time-normalised to a standardised 12-min exposure to account for differences in playing duration between players. Accumulated (absolute) values represent total load across actual playing time, whereas relative values were expressed per minute of effective playing time.

### Measurements

Data collection focused on external training load variables. These were obtained using the Polar Team Pro GPS (Polar Electro GmbH, Büttelborn, Germany), which recorded data at a sampling frequency of 10 Hz. The validity and reliability of the Polar Team Pro system have been examined in previous team-sport research, demonstrating acceptable inter-unit reliability and moderate-to-good validity for distance and speed measurements under outdoor conditions [7, 19].

External training load was assessed through four variables: total distance covered (TD), number of accelerations (ACC), number of decelerations (DEC) and high speed running (HSR) distance. TD was measured in metres (m) and reflected the cumulative distance a player covered during each 12-min session. Accelerations and decelerations were defined as the number of instances in which a player exceeded a threshold of ± 2.0 m∙s^−2^, while HSR was defined as runs above 19.8 km∙h^−1^. All data were manually extracted from the Polar Team Pro system and segmented to include only the relevant 12-min periods corresponding to active game time. The load values were then averaged across the five sessions for each participant and categorised based on whether the player's team won, drew, or lost each 12-min period.

### Statistical analysis

All statistical analyses were performed using IBM SPSS Statistics (version 31; IBM Corp., Armonk, NY, USA). Descriptive statistics are presented as mean ± standard deviation (SD). The level of statistical significance was set at *p* < 0.05. To examine differences in external load variables across match outcomes (win, draw, loss), a one-way repeated measures analysis of variance (ANOVA) was conducted for each dependent variable, total distance [TD], accelerations [ACC], decelerations [DEC]) and HSR. Match outcome (three levels: win, draw, loss) was treated as a within-subject factor. The assumption of normality was assessed using Shapiro–Wilk tests and visual inspection of Q–Q plots. Sphericity was evaluated using Mauchly’s test. When the assumption of sphericity was violated, Greenhouse–Geisser corrections were applied to adjust the degrees of freedom. Where significant main effects were observed, Bonferroni-adjusted pairwise comparisons were performed to identify differences between conditions. Effect sizes were reported as partial eta squared (η^2^p) and interpreted as small (0.01), moderate (0.06), and large (0.14). Where significant or relevant pairwise comparisons were examined, Bonferroni-adjusted mean differences with 95% confidence intervals were reported. Pairwise effect sizes were calculated as Cohen’s dz. Pairwise effect sizes were interpreted using conventional Cohen’s d thresholds, where values around 0.20, 0.50, and 0.80 were considered small, moderate, and large, respectively. These thresholds were used as descriptive guidelines rather than strict cut-offs.

## Results

Across all external load variables, a consistent pattern was observed between match outcomes in the rolling format. Descriptive values demonstrated that total (absolute) load variables were highest during win conditions and lowest during loss conditions, with draw values consistently positioned between these two. This pattern was evident for total distance, accelerations, decelerations, and HSR. In contrast, when these variables were expressed relative to playing time, no statistically significant differences were identified between win, draw, and loss conditions. To provide a concise statistical overview of the main effects, Table 1 presents the F-values, *p*-values, and partial eta squared (η^2^p) for all total and relative variables. Effect sizes ranged from small to large (η^2^p = 0.03–0.52), with the largest effects observed for total distance and decelerations.

**Table 1:** Repeated measures ANOVA results across SSG outcomes ^a^ greenhouse–geisser correction applied due to violation of sphericity

Variable	df (effect, error)	F	p	η^2^p	Sphericity
Total Distance	1.31, 22.34^a^	18.04	<.001	.52	GG corrected
Distance per minute	2, 34	1.57	.223	.09	Assumed
Accelerations (total)	2, 32	6.05	.006	.27	Assumed
Accelerations per minute	2, 32	0.49	.620	.03	Assumed
Decelerations (total)	2, 32	8.11	.001	.34	Assumed
Decelerations per minute	2, 32	1.72	.196	.10	Assumed
HSR (total)	1.37, 21.93^a^	3.74	.055	.19	GG corrected
HSR per minute	2, 32	0.45	.642	.03	Assumed

**Fig. 2:**
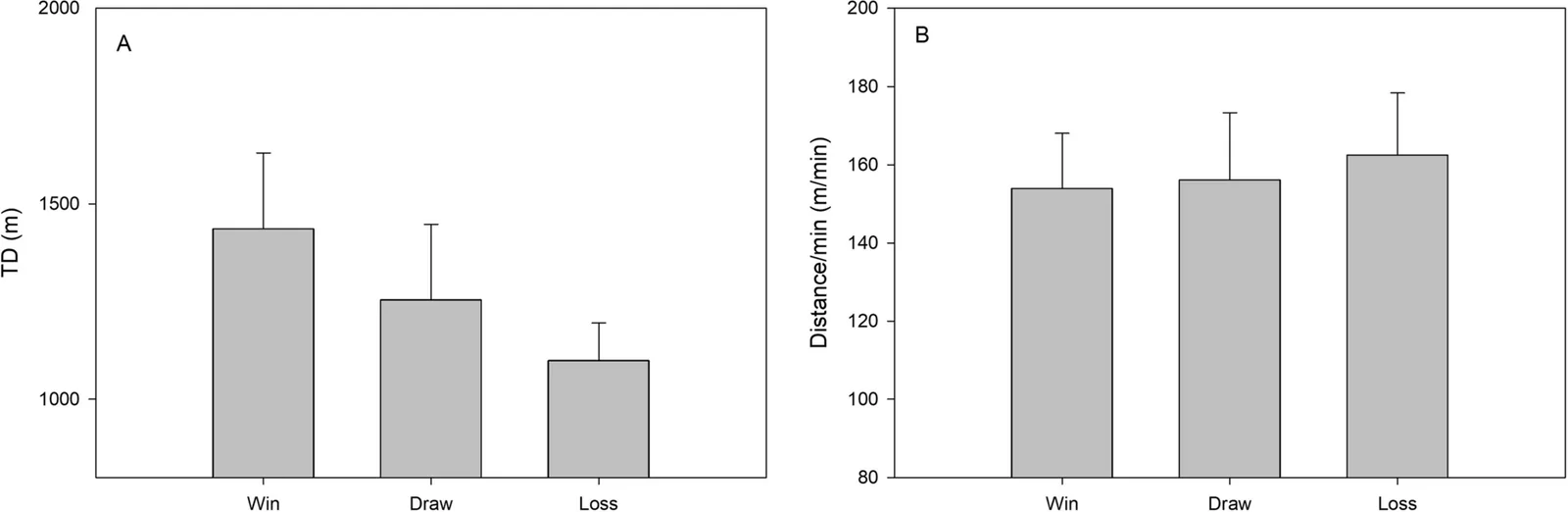
Total and relative distance across match outcomes in rolling small-sided games. **A** Total distance (TD, m) and **B** distance per minute (m·min⁻^1^) during win, draw, and loss conditions. Data are presented as mean ± standard deviation. A significant effect of match outcome was observed for total distance, with a progressive reduction from win to loss conditions, whereas no significant differences were found for distance per minute

For total distance, a significant main effect of match outcome was observed after applying the Greenhouse–Geisser correction due to violation of sphericity (*p* < 0.001). Total distance decreased progressively from win to draw to loss conditions, with a significant linear trend indicating a systematic reduction across outcomes. This pattern is illustrated in Fig. 2A. However, when total distance was normalised to playing time, no significant effect of match outcome was found (*p* > 0.05), as presented in Fig. 2B. Although players accumulated greater overall distance during win conditions, distance covered per minute remained comparable across outcomes.

**Fig. 3:**
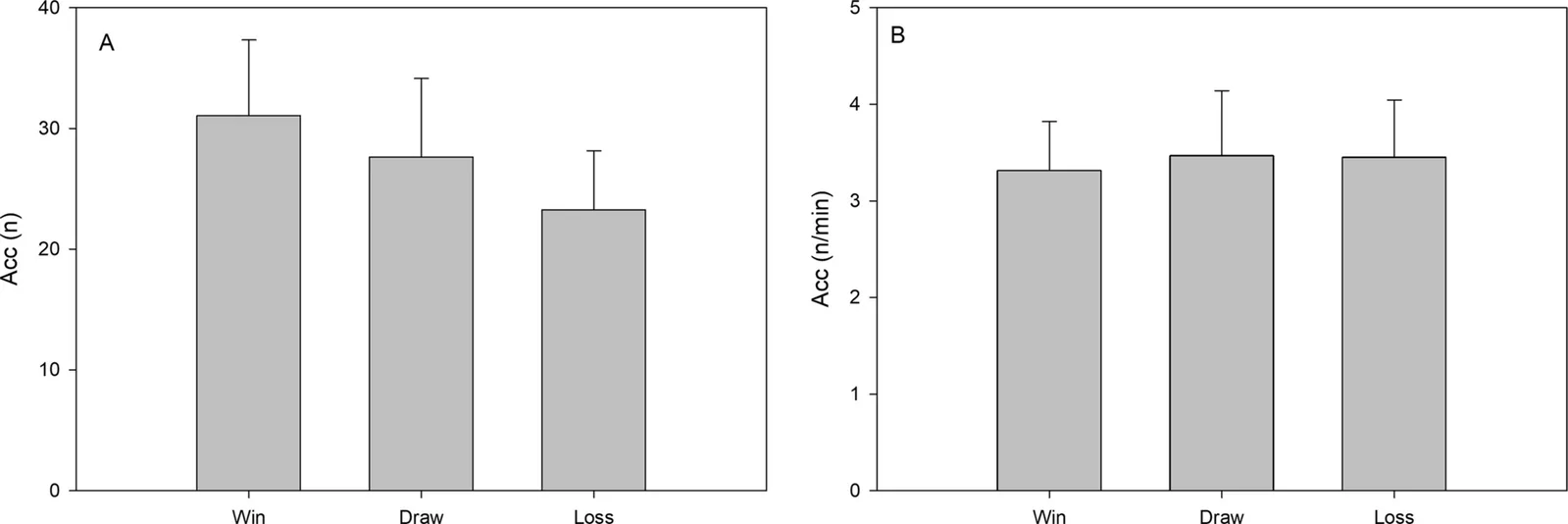
Total and relative accelerations by match outcome. **A** Total accelerations and **B** accelerations per minute across win, draw, and loss conditions. Values are mean ± SD. Total accelerations differed significantly between outcomes, with a reduction from win to loss conditions, whereas no significant differences were observed for accelerations per minute

A similar pattern was observed for accelerations. Total accelerations differed significantly between match outcomes (*p* = 0.006), with the highest values recorded during win conditions and the lowest during loss conditions. A significant linear trend confirmed a progressive reduction across outcomes. This is visualised in Fig. 3A. In contrast, accelerations per minute did not differ significantly between conditions (*p* > 0.05), as shown in Fig. 3B.

**Fig. 4:**
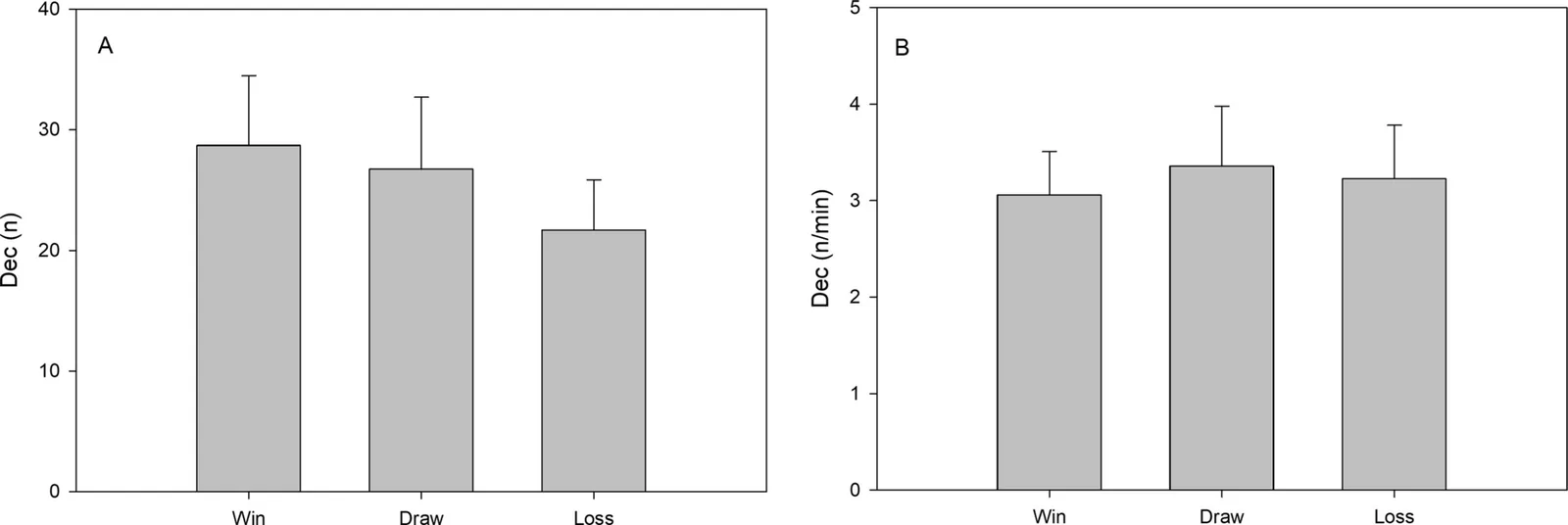
Total and relative decelerations by match outcome. **A** Total decelerations and **B** decelerations per minute across win, draw, and loss conditions. Values are mean ± SD. Total decelerations differed significantly between outcomes, with a progressive reduction from win to loss conditions, while relative decelerations did not differ significantly

Total decelerations also demonstrated a significant main effect of match outcome (*p* = 0.001), again with a clear linear reduction from win to loss conditions (Fig. 4A). However, when expressed relative to playing time, no significant differences were observed (*p* > 0.05), as illustrated in Fig. 4B. Relative decelerations did not differ significantly between outcomes.

**Fig. 5:**
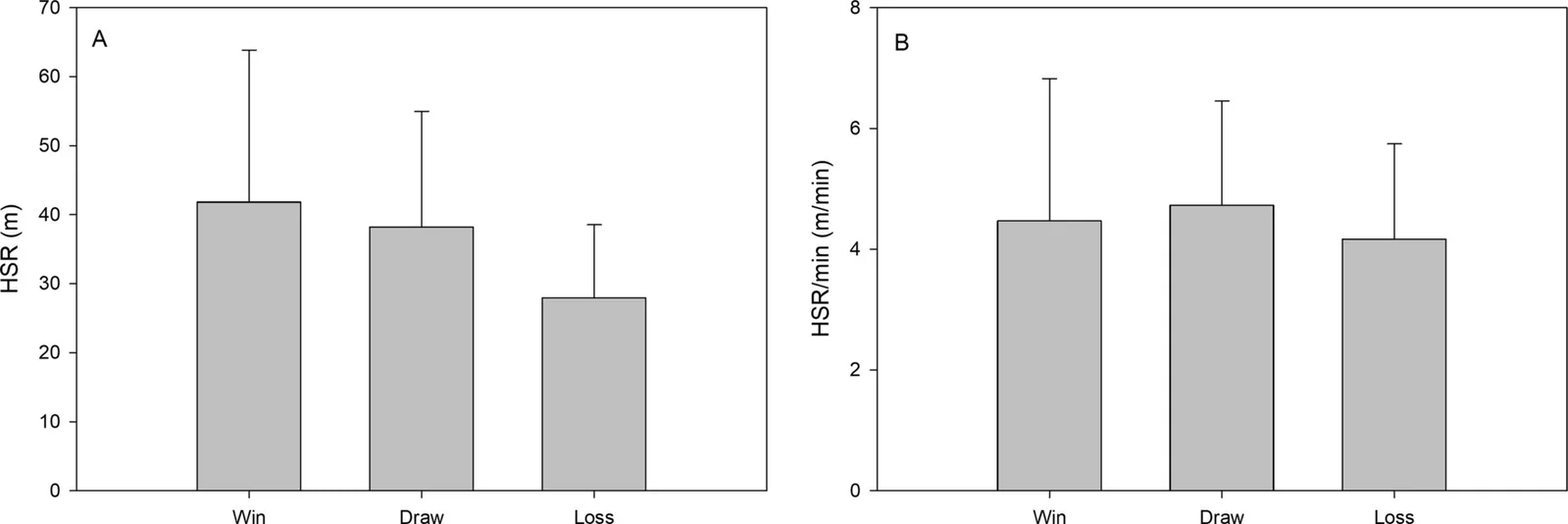
Total and relative high-speed running by match outcome. **A** Total high-speed running (HSR, m) and **B** HSR per minute (m·min⁻^1^) across win, draw, and loss conditions. Values are mean ± SD. The Greenhouse–Geisser-corrected omnibus effect for total HSR did not reach statistical significance, although descriptive values decreased from win to loss conditions. No significant differences were observed for HSR per minute

For total HSR, Mauchly’s test indicated violation of sphericity, and Greenhouse–Geisser correction was applied. The corrected main effect did not reach statistical significance (*p* = 0.055), although the descriptive pattern indicated a reduction from win to draw to loss conditions (Fig. 5A). As shown in Table 2, Bonferroni-adjusted pairwise comparisons should be interpreted cautiously because the corrected omnibus effect was not statistically significant. Despite this trend in accumulated HSR, no significant differences were observed for HSR per minute (*p* > 0.05), as presented in Fig. 5B.

**Table 2:** Bonferroni-adjusted pairwise comparisons between match outcomes for accumulated and time-normalised external load variables

Variable	Comparison	Mean difference	95% CI	Bonferroni-adjusted p	Cohen’s dz
Total distance (m)	Win vs draw	211.44	8.32 to 414.57	.040	0.65
Total distance (m)	Win vs loss	350.33	209.18 to 491.48	<.001	1.55
Total distance (m)	Draw vs loss	138.89	30.32 to 247.46	.010	0.80
Distance per minute (m·min⁻^1^)	Win vs draw	−1.78	−15.56 to 12.01	1.000	−0.08
Distance per minute (m·min⁻^1^)	Win vs loss	−7.56	−20.18 to 5.07	.391	−0.37
Distance per minute (m·min⁻^1^)	Draw vs loss	−5.78	−14.22 to 2.66	.260	−0.43
Accelerations (total)	Win vs draw	3.82	−3.05 to 10.70	.470	0.36
Accelerations (total)	Win vs loss	7.59	1.70 to 13.48	.010	0.84
Accelerations (total)	Draw vs loss	3.77	−0.70 to 8.23	.116	0.55
Accelerations per minute (n·min⁻^1^)	Win vs draw	−0.15	−0.78 to 0.47	1.000	−0.16
Accelerations per minute (n·min⁻^1^)	Win vs loss	−0.19	−0.73 to 0.35	1.000	−0.23
Accelerations per minute (n·min⁻^1^)	Draw vs loss	−0.04	−0.51 to 0.43	1.000	−0.06
Decelerations (total)	Win vs draw	2.29	−3.68 to 8.27	.959	0.25
Decelerations (total)	Win vs loss	7.18	2.40 to 11.95	.003	0.97
Decelerations (total)	Draw vs loss	4.88	1.34 to 8.43	.006	0.89
Decelerations per minute (n·min⁻^1^)	Win vs draw	−0.30	−0.83 to 0.23	.436	−0.37
Decelerations per minute (n·min⁻^1^)	Win vs loss	−0.17	−0.57 to 0.23	.818	−0.27
Decelerations per minute (n·min⁻^1^)	Draw vs loss	0.13	−0.24 to 0.51	1.000	0.23
High-speed running (m)	Win vs draw	5.06	−13.61 to 23.72	1.000	0.18
High-speed running (m)	Win vs loss	14.94	−0.07 to 29.95	.051	0.65
High-speed running (m)	Draw vs loss	9.88	0.44 to 19.32	.039	0.68
High-speed running per minute (m·min⁻^1^)	Win vs draw	−0.14	−2.03 to 1.74	1.000	−0.05
High-speed running per minute (m·min⁻^1^)	Win vs loss	0.41	−1.31 to 2.12	1.000	0.15
High-speed running per minute (m·min⁻^1^)	Draw vs loss	0.55	−0.55 to 1.64	.599	0.32

Positive mean differences indicate higher values in the first-listed outcome condition. Pairwise comparisons were Bonferroni-adjusted. Cohen’s dz was calculated as the paired-comparison t-value divided by the square root of n. Analyses were based on *n* = 18 for total distance and distance per minute, and *n* = 17 for accelerations, decelerations, and high-speed running variables. The pairwise comparisons for high-speed running should be interpreted cautiously because the Greenhouse–Geisser-corrected omnibus effect did not reach statistical significance

Bonferroni-adjusted pairwise comparisons are presented in Table 2. For accumulated external load, total distance differed significantly between all match outcomes, with higher values for win than draw and loss, and higher values for draw than loss. Accelerations differed significantly between win and loss, while decelerations differed significantly between win and loss and between draw and loss. No significant pairwise differences were observed for any time-normalised variable. Pairwise comparisons for accumulated HSR should be interpreted cautiously because the Greenhouse–Geisser-corrected omnibus effect did not reach statistical significance.

## Discussion

The present study investigated whether match outcome in a rolling three-team small-sided game format influences external training load. The principal finding was that match outcome acts as a volume amplifier rather than an intensity modifier. Importantly, this effect is not driven by winning, but by the extended playing time imposed by the winner-stays rule. Specifically, winning significantly increased accumulated external load (TD, ACC, and DEC), whereas relative intensity (per-minute load) remained unchanged across all outcomes. This demonstrates that outcome-related differences in physical load are driven by playing exposure. Consequently, the winner-stays rule effectively manipulates the total mechanical dose without altering the instantaneous physical demands of the game. This finding aligns with previous research showing that structural constraints such as pitch size and work-to-rest ratios primarily influence accumulated load within SSGs through changes in exposure time rather than per-minute intensity [8, 13, 20]. The present findings extend this literature by demonstrating that not only static task constraints, but also dynamic competitive mechanisms embedded within the rules, can modulate accumulated load.

Existing research has established that manipulating pitch size, player number, and work-to-rest ratios significantly alters external load in SSGs [8, 13, 20]. Traditionally, load variation is conceptualized as a direct result of these static task constraints. For example, larger relative pitch areas increase high-speed running and total distance, while reduced player numbers increase individual involvement and physiological strain. This is supported by Dellal et al. (2012) who showed that rule modifications can elevate high-intensity actions [12]. Collectively, this literature has conceptualized load variation as primarily driven by task design. However, the present findings indicate that even when structural constraints remain, the rule-based rotation mechanism introduces systematic load asymmetries. In the rolling format, the winner-stays rule functions as a dynamic constraint that redistributes playing time between teams. Match outcome should therefore be understood as an emergent variable through which this rule operates, rather than a primary causal driver. Consequently, differences in accumulated load arise due to variations in exposure duration inherent to the rotation rule.

Importantly, the absence of differences in per-minute load suggests that players maintain consistent physical output while actively participating, regardless of match outcome. This finding is consistent with prior findings indicating that players tend to maintain relatively stable physical output during active play, regardless of contextual constraints [8]. Specifically, our results parallel those of Branquinho et al. [17], who demonstrated that altering recovery durations between SSG bouts primarily manipulates total accumulated external load while keeping per-minute movement intensities remarkably stable. In contrast, our findings diverge from literature examining full competitive match-play, where contextual variables such as match status or scoreline often lead to changes in relative intensity and pacing profiles [16]. While players in full matches may adjust effort based on tactical demands or psychological pressure, this rolling SSG format appears to constrain pacing behavior. However, alternative explanations should also be considered. Differences in tactical behaviour, spatial organization, or team strategies between winning and losing teams may have influenced the observed external load patterns. This is consistent with previous research demonstrating that contextual and tactical factors, including match status and pacing strategies, can influence physical responses in team sports [15–17]. Structural exposure rules, rather than psychological momentum, dictated the load Consequently, competitive success did not enhance instantaneous intensity; it simply extended time under load. This reinforces the distinction between external volume and relative intensity. From a mechanistic perspective, the rolling format functions as an exposure amplifier, rewarding success with prolonged play and increasing cumulative mechanical load without necessarily altering physiological or neuromuscular output per unit time.

The consistent linear reduction observed across total distance, accelerations, and decelerations further strengthens this interpretation. Similar linear dose–response relationships have been reported when relative pitch area increases [13] or when sprint capacity influences external load expression [14]. However, unlike these studies, the present findings demonstrate that a competitive mechanism embedded within the training structure can produce comparable linear load modulation. Furthermore, this systematic modulation is likely intensified by the altered work-to-rest ratios inherent in the winner-stays format. While losing teams receive full recovery between bouts, winning teams are forced into repeated exposures with truncated rest intervals. Because internal load was not measured in the present study, the physiological consequences of repeated consecutive bouts remain speculative. However, the shorter recovery periods experienced by successful teams may have increased internal physiological strain despite similar per-minute external load. Future studies should include heart rate, blood lactate, RPE, or other internal load markers to examine whether rolling winner-stays formats produce a dissociation between external and internal load.

This implies that competitive structure represents an additional, underexplored constraint within SSG design. Although total high-speed running did not reach statistical significance after Greenhouse–Geisser correction, the descriptive pattern and moderate effect size are consistent with prior literature reporting greater variability in high-speed metrics [18]. High-speed actions are inherently more sensitive to contextual variability and tactical spacing. Nevertheless, the same volume–intensity separation was observed, reinforcing the robustness of the overall pattern. Taken together, these findings indicate that rolling SSG formats create systematic asymmetries in accumulated training load based on competitive outcome. This adds an important layer to existing SSG literature, which has primarily examined how structural constraints influence intensity. The present study demonstrates that competitive structure can selectively manipulate volume while maintaining stable per-minute intensity. Consequently, volume and intensity must be analytically distinguished when evaluating the training demands of rolling formats.

### Strength and limitations

This study has several notable strengths. It was conducted under ecologically valid training conditions, ensuring that the findings reflect real-world football practice. The focus on the competitive “winner-stays-on” mechanism represents a novel contribution to small-sided game (SSG) research, extending knowledge beyond traditional structural variables such as pitch size and player number. The repeated-measures design across five sessions increases internal reliability, and the combined use of absolute and relative load metrics provides a nuanced understanding of how match outcome affects training load. Finally, the use of validated GPS technology strengthens the accuracy of the external load measurements, and the clear linear patterns observed across conditions highlight the robustness of the findings.

However, the present findings should be interpreted considering certain limitations. The study was conducted within a single team, which may limit generalizability to other competitive levels. Although the rolling format was structurally standardised, tactical behaviour and spatial organization were not directly quantified, and subtle behavioural adaptations between winning and losing teams cannot be excluded. Although teams were formed by the coaching staff to balance playing positions and perceived playing level, team strength was not statistically matched. Therefore, residual differences in team quality may have influenced match outcomes, playing time, and accumulated external load. Additionally, while external load was comprehensively assessed, physiological internal load markers were not included, which limits the ability to observe the direct metabolic cost of the exposure asymmetries. Technical and tactical behaviours were not quantified, and differences in playing style between successful and unsuccessful teams may partly explain load distributions. Furthermore, psychological factors such as motivation and effort regulation were not measured and may influence external load expression across winning and losing conditions. Finally, the study examined acute responses within sessions; the long-term implications of repeated exposure asymmetries remain unknown. Future research should examine whether consistent outcome-driven differences in accumulated volume influence adaptation, fatigue, or injury risk over time.

### Practical applications

From an applied perspective, the findings suggest that rolling SSG formats are effective for maintaining stable per-minute physical intensity across teams. However, they inherently create unequal accumulated external load depending on competitive success. Coaches implementing rolling formats may therefore consider monitoring total exposure time, particularly in repeated training contexts where systematic differences in team performance could emerge. If the objective is to ensure equitable load distribution across players, practitioners could adjust rotation rules, impose minimum playing quotas, or equalize exposure across intervals to avoid unintended underloading of losing teams. Conversely, if the goal is to progressively overload high-performing units while maintaining intensity, rolling formats could represent a practical mechanism for doing so without altering task constraints such as pitch size or player number. Coaches may consider using rolling SSGs on days where higher mechanical volume is intended, such as mid-week overload days, since the format may increase cumulative load for successful players without necessarily altering the intensity demands. Conversely, such formats could be less appropriate on tapering days where load equality and controlled volume are prioritized. Since accumulated load varies by match outcome, practitioners may need to account for competitive success when interpreting weekly or monthly load data. Failure to do so may lead to differences in accumulated exposure between players, which could have implications for load management. However, outcomes such as injury risk and readiness were not assessed in the present study. Finally, these results underscore the importance of reporting both absolute and time-normalised load metrics. Interpreting only total load could lead to the erroneous conclusion that intensity differs between competitive conditions. Integrating both volume and intensity metrics may allow for a more accurate evaluation of training stimulus and support more precise load management strategies.

## Conclusion

In conclusion, match outcome within a rolling small-sided game format significantly influenced accumulated external load but did not affect relative intensity per playing time. Across total distance, accelerations, and decelerations, accumulated metrics decreased from win to loss conditions, whereas time-normalised measures remained stable. Total HSR showed a similar descriptive pattern but did not reach statistical significance. These findings are consistent with the broader literature on SSG design, which emphasizes the role of task constraints in shaping training load responses, but indicate that the observed increase in accumulated load among winning teams is driven by extended playing time inherent to the winner-stays rule, rather than the act of winning itself. Importantly, match outcome should not be interpreted as the primary causal factor, but rather as an emergent outcome through which the winner-stays rule redistributes playing time and thereby regulates accumulated load. The winner-stays mechanism increases playing duration for successful teams, thereby elevating accumulated mechanical load without altering the instantaneous physical demands imposed by the game. This distinction reinforces the importance of analytically separating volume from intensity when evaluating training demands. From an applied perspective, rolling formats inherently produce asymmetrical exposure based on competitive outcome. This mechanism may therefore be considered when designing training sessions, either to promote equitable load distribution or to selectively increase mechanical volume in higher-performing players. Overall, recognising how competitive dynamics influence accumulated load may support more precise interpretation of training stimuli and more informed monitoring of player workload in football-specific contexts.

## Data Availability

The datasets generated and analysed during the current study are not publicly available due to privacy and ethical restrictions but are available from the corresponding author on reasonable request.
